# DP-CARE: a differentially private classifier for mental health analysis in social media posts

**DOI:** 10.3389/fdgth.2025.1709671

**Published:** 2025-12-11

**Authors:** Dimitris Karpontinis, Efstathia Soufleri

**Affiliations:** Archimedes, Athena Research Center, Athens, Greece

**Keywords:** mental health, LLM, stress detection, privacy, differential privacy

## Abstract

**Introduction:**

Mental health NLP models are increasingly used to detect psychological states such as stress and depression from user-generated social media content. Although transformer based models such as MentalBERT achieve strong predictive performance, they are typically trained on sensitive data, raising concerns about memorization and unintended disclosure of personally identifiable information.

**Methods:**

We propose DP-CARE, a simple yet effective privacy-preserving framework that attaches a lightweight classifier to a frozen, domain-specific encoder and trains it using Differentially Private AdamW (DP-AdamW). This approach mitigates privacy risks while maintaining computational efficiency.

**Results:**

We evaluate DP-CARE on the Dreaddit dataset for stress detection. Our method achieves competitive performance, with an F1 score of 78.08% and a recall of 88.67%, under a privacy budget of ε ≈ 3.

**Discussion:**

The results indicate that lightweight, differentially private fine-tuning offers a practical and ethical approach for deploying NLP systems in privacy-sensitive mental health contexts. DP-CARE demonstrates that strong predictive performance can be retained while significantly reducing privacy risks associated with training on sensitive user data.

## 1 Introduction

Natural language processing (NLP) for mental health is a rapidly evolving field, with growing interest in using language models to detect symptoms such as depression, stress, and anxiety from user-generated text [1, 2]. The availability of large-scale social media platforms like Reddit and Twitter [3], where individuals often share emotional and psychological experiences, has enabled researchers to train models capable of identifying mental health signals from text with high accuracy.

However, these models are typically trained on sensitive and potentially identifiable data, raising significant privacy concerns. Posts may contain personal disclosures, trauma narratives, or stigmatizing content that, if memorized or leaked by a model, could result in harm, ethical violations, or breaches of regulatory standards such as HIPAA or GDPR [4]. These risks are magnified in real-world or resource-limited deployments—such as mobile mental health applications or digital therapy tools—where the consequences of a privacy breach may directly affect vulnerable individuals [5–9].

Training on user-generated mental health data introduces a unique set of privacy risks [10–18]. Even when models are designed to limit updates to downstream components—such as lightweight classifiers operating on frozen encoder outputs—they may still expose sensitive patterns present in the training data. This is especially concerning in mental health contexts, where personal disclosures often contain identifying or emotionally charged language. Without explicit safeguards, models remain susceptible to privacy attacks and unintended leakage, underscoring the need for methods that offer formal and provable protection.

Differential Privacy (DP) offers a principled solution by bounding the contribution of any individual training example to the learned parameters [19, 20]. Optimizers such as Differentially Private AdamW (DP-AdamW), implemented through frameworks like Opacus [21], enable privacy-preserving training by clipping per-sample gradients and injecting calibrated noise during optimization. While DP has been widely studied in general-purpose machine learning, its application to mental health NLP remains limited—largely due to the computational challenges of privacy-aware training and the heightened sensitivity of the underlying data.

In this work, we introduce **DP-CARE**, a simple yet effective framework for privacy-preserving mental health text classification. Instead of fine-tuning the full model, DP-CARE trains only a lightweight classifier on top of a frozen, domain-specific encoder (MentalBERT) [22], using DP-AdamW to enforce differential privacy. This design ensures computational efficiency and stronger privacy guarantees while retaining competitive task performance.

Our main contributions are as follows:

- We propose a lightweight and modular privacy-preserving framework, DP-CARE, that applies DP-AdamW to train only a classifier on top of a frozen mental health-specific encoder.
- We demonstrate that this simple architecture achieves strong performance on the Dreaddit stress detection task (F1 = 78.08%, Recall = 88.67%) while maintaining a formal privacy budget of ε≈3.
- We show that DP-CARE offers a practical and deployable alternative to full-model fine-tuning, making it especially well-suited for real-world, resource-constrained, and privacy-sensitive applications.

## 2 Why privacy matters in mental health NLP

**Figure 1:**
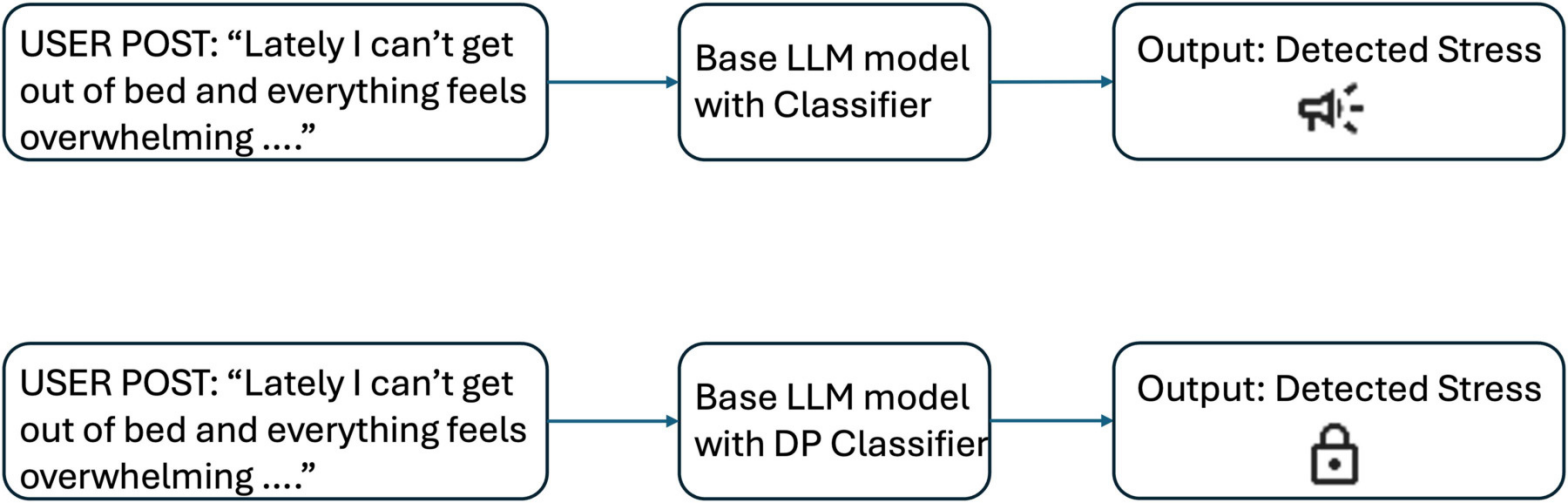
Illustration of privacy risks in mental health NLP. **Top:** Without differential privacy, the classifier may memorize unique user phrases, posing a risk of privacy leakage. **Bottom:** With differential privacy (DP-AdamW), memorization and leakage risk are reduced. “Speaker” and “lock” icons from Google Material Icons, licensed under Apache License 2.0.

Privacy is a foundational concern in mental health NLP due to the nature of the data and the high stakes involved. Unlike domains such as product reviews or general news, mental health content frequently includes deeply personal narratives involving trauma, relationships, self-harm, or psychiatric conditions [23]. These disclosures, even when posted in public online spaces, are often not intended for algorithmic consumption. As a result, models trained on such data risk capturing and internalizing language that is both sensitive and uniquely identifying (Figure 1).

Recent studies have shown that large language models are capable of memorizing rare training examples [5, 6]. In mental health contexts, this raises the alarming possibility that private information—such as a user’s suicidal ideation or abuse history—could be surfaced through benign queries or adversarial attacks. Even anonymized datasets do not fully mitigate this risk, as the co-occurrence of symptom patterns or distinctive phrasing can serve as indirect identifiers.

Moreover, the use of mental health NLP [24] in real-world settings—such as chat-based therapy assistants, clinical decision support systems, or wellness monitoring tools—places additional constraints on privacy. These applications demand both accuracy and rigorous guarantees that personal information cannot be leaked or reconstructed. Traditional training pipelines, which focus solely on predictive performance, fail to meet this standard. They expose models to privacy attacks such as membership inference and model inversion, where adversaries can determine whether a particular user’s data was used in training or attempt to reconstruct sensitive inputs from model outputs [8].

To address these challenges, Differential Privacy (DP) provides a principled solution [19, 25]. By ensuring that the inclusion or exclusion of a single training example has a provably bounded effect on model parameters, DP offers strong protections against data leakage. Optimizers like DP-AdamW offers this guarantee by clipping individual gradients and adding calibrated noise during training. In mental health NLP, where the consequences of privacy breaches are both personal and severe, integrating DP into model training is not merely beneficial—it is essential for responsible deployment.

## 3 Related work

Prior work on mental health detection from social media has primarily focused on improving predictive accuracy. Domain-specific transformer models such as MentalBERT and MentalRoBERTa [1] have been trained on Reddit and Twitter posts annotated with mental health labels, achieving state-of-the-art performance on tasks such as depression and stress classification. However, these models do not account for the privacy risks associated with training on sensitive user-generated content.

Differential Privacy (DP), introduced by Dwork et al. [19], offers a mathematically rigorous framework for bounding the privacy loss incurred during model training. DP aware training algorithms, proposed by Abadi et al. [25], is the most widely adopted algorithm for training deep neural networks under DP constraints. More recent studies have highlighted the susceptibility of large language models to memorization and privacy leakage [26], as well as the trade-offs involved in applying DP-AdamW to transformer architectures such as BERT [27].

In the clinical NLP domain, models like ClinicalBERT [28], fine-tuned on electronic health records, have demonstrated improvements in tasks such as named entity recognition and medical concept extraction. Meanwhile, tools such as LIWC and behavioral analysis pipelines [29, 30] have long been used to extract mental health signals from linguistic patterns. Nonetheless, few of these approaches explicitly address privacy-preserving training, and even fewer explore efficient alternatives to full-model fine-tuning, such as training lightweight classifier modules under DP [25].

## 4 Methods

**Figure 2:**
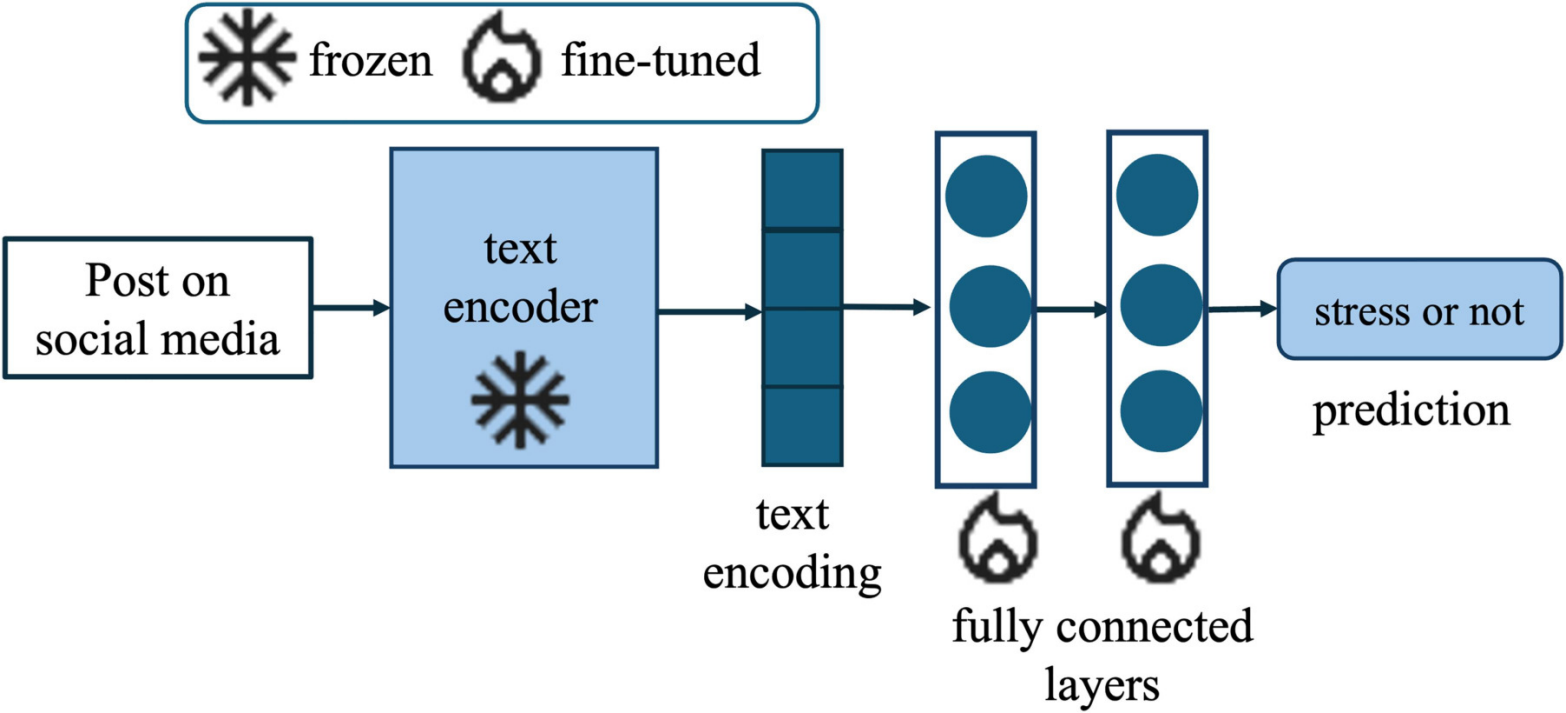
Overview of the DP-CARE architecture. A pre-trained domain-specific encoder (MentalBERT) [1] is frozen and used to extract contextual embeddings from input text. A lightweight classifier is attached to the text encoder output and trained using Differentially Private AdamW (DP-AdamW). Only the classifier parameters are updated, ensuring both computational efficiency and formal privacy guarantees under differential privacy. “Snowflake” and “fire” icons from Google Material Icons, licensed under Apache License 2.0.

Our proposed framework, DP-CARE, is designed to balance privacy protection, computational efficiency, and classification performance in mental health text classification. The architecture consists of a frozen transformer-based encoder and a lightweight classifier trained with differential privacy (Figure 2).

### 4.1 Base encoder: frozen mentalBERT

We use a pre-trained domain-specific language model, MentalBERT [1], as the feature extractor. The encoder is frozen during training, meaning its parameters remain unchanged. This design choice preserves the rich mental health-specific representations learned during pretraining, while also significantly reducing the number of trainable parameters, memory usage, and training time. The encoder produces contextualized embeddings from each input text, from which we extract the text encoding.

### 4.2 Classifier

A lightweight feedforward classifier is attached to the frozen encoder. It consists of a single or multi-layer perceptron (MLP) with a softmax output layer for binary classification (stress vs. no stress). The classifier is the only trainable component of the model and is responsible for mapping the fixed encoder representations to stress labels.

### 4.3 Differentially private training

To ensure privacy-preserving learning, we train the classifier using Differentially Private AdamW (DP-AdamW) [25]. DP-AdamW operates by clipping per-example gradients to a fixed norm and adding calibrated Gaussian noise to the aggregated gradient update. This process ensures that the model’s behavior is not unduly influenced by any single training example, thereby providing a formal guarantee of (ε,δ)-differential privacy.

We implement DP-AdamW using the Opacus library [21]. The encoder is frozen and excluded from the DP computation graph, allowing the privacy budget to be spent exclusively on the classifier parameters. This design minimizes privacy cost while leveraging the strength of pre-trained mental health-specific features.

### 4.4 Privacy-efficiency trade-off

By decoupling the classifier from the encoder and applying DP-AdamW only to the classifier, DP-CARE achieves a favorable privacy—efficiency trade-off. This approach reduces the computational burden and avoids the instability commonly associated with full-model DP training on large transformers. As a result, DP-CARE is particularly well-suited for deployment in low-resource or privacy-sensitive settings, such as mobile health applications or clinical screening tools.

## 5 Results

### 5.1 Dataset

We evaluate DP-CARE on the Dreaddit dataset, a benchmark for stress detection in social media posts [31].^1^ Dreaddit is a Reddit-based corpus composed of user-generated posts labeled as either stressful or non-stressful. The data is collected from ten subreddits spanning a range of domains, including mental health—related communities such as r/anxiety and r/PTSD, as well as general life topics such as r/family, r/work, and r/relationships. Posts are thematically grouped into categories related to interpersonal conflict, mental illness, and financial need, reflecting real-world stressors faced by users.

The final dataset consists of 3,553 posts, divided into predefined training, validation and test splits as provided by the dataset authors. Each post is accompanied by a binary label indicating the presence or absence of psychological stress. We follow standard preprocessing procedures: text is tokenized using the MentalBERT tokenizer, and all inputs are truncated to a maximum sequence length of 512 tokens [1].

Dreaddit is well-suited for evaluating stress classifiers in realistic and noisy online environments, and its focus on multiple high-stakes domains makes it an appropriate testbed for assessing privacy-preserving NLP models in mental health contexts.

### 5.2 Experimental setup

We use MentalBERT as a frozen encoder and attach a single-layer feedforward classifier with a softmax output for binary stress classification. The classifier is trained under two regimes: standard AdamW optimization as a non-private baseline, and DP-AdamW, a differentially private variant implemented via the Opacus library, for privacy-preserving training. To ensure differential privacy, we clip each per-example gradient to an $\ell_{2}$ norm of 1.0 and add Gaussian noise with a noise multiplier of 1.1 to the aggregated gradients. In the non-dp setting, gradient clipping is also set to 1.0. We train both models for 10 epochs using a batch size of 64, and set the privacy accountant’s target parameter to $\delta =10^{-5}$. Under these conditions, the final privacy budget, computed using Rényi Differential Privacy accountant (RDP), is approximately ε≈3. Because the encoder is frozen, all privacy expenditure is attributed solely to the classifier parameters, substantially reducing both the privacy risk and the computational overhead. Table 1 summarizes the key hyperparameter settings. For reproducibility, we provide pseudocode of the DP-CARE training loop in Appendix 1.

**Table 1:** Training hyperparameters and differential privacy settings used for fine-tuning DP-CARE on the Dreaddit dataset.

Parameter	Value
Optimizer	DP-AdamW (Opacus)
Noise multiplier	1.1
Max gradient norm	1.0
Privacy budget (ε, δ)	(3.0, $10^{-5}$)
Batch size	64
Epochs	10
Learning rate	0.01

The non-private model is trained with a learning rate of $10^{-2}$ and uses a linear learning rate scheduler with warmup. In contrast, the differentially private model uses a smaller learning rate of $10^{-3}$ and does not employ a scheduler. The AdamW optimizer was selected in both cases to promote faster convergence. All experiments are conducted on a single NVIDIA Tesla T4 GPU (16 GB memory) with early stopping based on validation loss.

### 5.3 Evaluation metrics

We report F1 score and recall on the test set. All results are averaged over four random seeds to reduce variance.

### 5.4 Results and analysis

We report classification performance in terms of recall and F1 score, averaged over four random seeds. Results are summarized in Table 2. To isolate the effect of differential privacy, both models share an identical architecture and training configuration, using a frozen MentalBERT encoder and the same classifier design and hyperparameters; the only difference is that DP-CARE employs DP-AdamW for private optimization of the classifier, while the baseline uses the standard AdamW optimizer without privacy noise or gradient clipping for training the classifier. The non-private classifier achieves strong performance across all metrics, with an F1 score of 80.58%. Note that the non-private classifier exhibits zero variance across seeds because the architecture is frozen, the classifier is small, and the training dynamics are deterministic under our fixed initialization and data-ordering scheme. When training the classifier using DP-AdamW, the model retains competitive overall performance with an F1 score of 78.08%. Interestingly, the DP-trained model achieves substantially higher recall (88.67%), suggesting that it is more sensitive to detecting the positive class (stressful posts), even at the cost of lower precision. This shift may be attributed to the regularization effect of DP-AdamW, which introduces noise into the training process and discourages overfitting. These findings illustrate a common pattern in differentially private training: while overall precision may drop, the model may become more inclusive in its predictions, which can be beneficial in high-stakes applications where false negatives (missed stressful posts) are riskier than false positives. Crucially, the DP-trained model achieves this performance while maintaining a formal privacy guarantee of ε≈3, demonstrating the feasibility of privacy-preserving training in sensitive mental health applications.

**Table 2:** Classification performance (Accuracy, Recall, and F1 score) on the Dreaddit dataset, averaged over four random seeds. Both models use an identical frozen MentalBERT encoder and trainable classifier architecture. DP-CARE applies DP-AdamW with noise multiplier = 1.1, max grad norm = 1.0, ε=2.85, and $\delta =10^{-5}$ to the classifier parameters, while the baseline uses the same training setup without differential privacy.

Model	Recall	F1 score
Frozen MentalBERT + Non-private classifier (Baseline)	81.99%±0.00	80.58%±0.00
DP-CARE (DP-AdamW, Ours)	88.67%±1.07	78.08%±0.24

### 5.5 Computational efficiency for DP and non-DP finetuned classifiers

We evaluated computational efficiency using a single NVIDIA Tesla T4 GPU (16 GB memory) with identical hyperparameters for both private and non-private fine-tuning (epochs = 10, batch size = 64, learning rate = 0.01). Compared to the non-private model, DP-CARE trained with DP-AdamW (noise multiplier = 1.1, max gradient norm = 1.0, $\delta =10^{-5}$) introduced only a minor runtime overhead of approximately 1.3% (378.6 s vs. 373.8 s total training time) and a moderate increase in peak GPU memory usage (1.23 GiB vs. 0.96 GiB). The increase in memory stems from per-sample gradient computations required for differential privacy accounting, while the small runtime overhead reflects the additional noise injection and gradient clipping steps.

Training times were measured using wall-clock time from the start to the end of model fine-tuning, and GPU memory usage was tracked via torch.cuda.max_memory_allocated().

Privacy accounting across epochs is reported in Table 3, where ε increases from 1.13 at epoch 1 to 2.85 at epoch 10.

**Table 3:** Compute profile and privacy accounting using a Tesla T4 GPU. DP-CARE adds only ≈1% runtime overhead relative to non-private fine-tuning while providing formal differential privacy guarantees.

Metric	Non-DP	DP-CARE
GPU type	Tesla T4 (16 GB)	Tesla T4 (16 GB)
Epochs/Batch size/LR	10/64/0.01	10/64/0.01
Steps per epoch	36	35
Total wall time (s)	373.8	378.6
Approx. steps/s	0.963	0.925
Peak GPU memory (GiB)	0.96	1.23
DP params (δ,σ,C)	n/a	$\left(10^{-5},\;1.1,\;1.0\right)$
ε (per epoch)	n/a	[1.13 → 2.85]

These findings indicate that DP-CARE preserves model accuracy while keeping the additional computational cost of differential privacy negligible, demonstrating that privacy preservation can be achieved without compromising training efficiency. Overall, DP-CARE maintains computational feasibility under privacy constraints.

### 5.6 Per-class and error analysis

To better understand class-wise behavior, Table 4 reports per-class Precision, Recall, and F1 Score (%) for the Dreaddit labels (0 = non-stress, 1 = stress), based on four DP-CARE training runs. The results reveal a clear trade-off introduced by differential privacy. For the non-stress class (label 0), DP-CARE maintains high precision (80.74% ± 0.85) but exhibits reduced recall (61.24% ± 1.83), leading to a lower F1 score (69.63% ± 0.95). This behavior indicates that, under DP noise, the model becomes more conservative when predicting the non-stress class, correctly identifying most of its positive predictions but failing to recover a substantial portion of true non-stress examples. In contrast, the stress class (label 1) shows the opposite pattern: recall improves considerably (86.67% ± 1.07), while precision decreases to 71.06% ± 0.74, resulting in an F1 score of 78.08% ± 0.24. This shift implies that DP-CARE becomes more sensitive to stress signals, capturing more true stress cases at the cost of additional false positives. From an application perspective, this behavior may be desirable: in mental-health screening, missing true stress cases (false negatives) is generally more concerning than flagging additional borderline cases. Thus, while DP-CARE introduces a class-specific trade-off, it increases recall where it is clinically most valuable.

**Table 4:** Per-class Precision, Recall, and F1 Score (%) on the Dreaddit test set, averaged over four DP-CARE runs. DP-CARE (DP-AdamW with noise multiplier = 1.1, max grad norm = 1.0, ε=2.85, $\delta =10^{-5}$) improves recall on the stress class while reducing recall on the non-stress class.

Class (Label)	Precision	Recall	F1 score
Non-stress (0)	80.74% ± 0.85	61.24% ± 1.83	69.63% ± 0.95
Stress (1)	71.06% ± 0.74	86.67% ± 1.07	78.08% ± 0.24

### 5.7 Full-model vs. classifier-only DP training

To further illustrate the computational advantages of our classifier-only privacy design, we compared the number of trainable and frozen parameters between full-model DP-AdamW and classifier-only DP-AdamW (DP-CARE) using MentalBERT as the base model architecture. Table 5 summarizes the overall parameter counts, while Table 6 provides a detailed module-level breakdown.

**Table 5:** Trainable vs. frozen parameters for full-model DP-AdamW and classifier-only DP-AdamW (DP-CARE) on MentalBERT as the base model. Training only the classifier head reduces the number of parameters participating in optimization by several orders of magnitude, leading to markedly lower computational and memory requirements.

Mode	Total params	Trainable params	Frozen params	Trainable %
Full-model DP-AdamW	109,483,778	109,483,778	0	100.0000%
Classifier-only DP (DP-CARE)	109,483,778	1,538	109,482,240	0.0014%

**Table 6:** Module-level parameter counts and trainability for full-model vs. classifier-only DP. In classifier-only DP (DP-CARE), all transformer layers are frozen and only the final classification head is updated, which drastically lowers computational demand.

Module	Params (total)	Trainable (full)	Trainable (head)	Share of total
Bert.encoder	85,054,464	85,054,464	0	77.6868%
Bert.embeddings	23,837,184	23,837,184	0	21.7723%
Bert.pooler	590,592	590,592	0	0.5394%
Classifier	1,538	1,538	1,538	0.0014%
Total	109,483,778	109,483,778	1,538	100.0000%

In the full-model configuration, DP-AdamW updates and privatizes all parameters of the transformer encoder, embeddings, and pooler layers, exceeding $10^{8}$ trainable parameters. This setup entails high computational cost since every parameter participates in gradient computation, clipping, and noise addition at each optimization step. In contrast, DP-CARE freezes the entire transformer and trains only the classifier head, which contains approximately $1.5\times 10^{3}$ parameters—over four orders of magnitude fewer than the full model. By avoiding gradient computation for the encoder, DP-CARE substantially reduces the number of operations and the associated memory footprint, enabling efficient fine-tuning under differential privacy while maintaining strong predictive performance.

### 5.8 Comparison with state of the art

To contextualize DP-CARE’s performance, we compare our results on the Dreaddit dataset against several pre-trained transformer-based models, including domain-specific and clinical adaptations. Table 7 reports the recall and F1 score for each model, along with our proposed method.

**Table 7:** Comparison with state-of-the-art models on the Dreaddit dataset, reported in terms of Recall and F1 score. Baseline results are taken from [1]. For DP-CARE, we used DP-AdamW with noise multiplier = 1.1, max grad norm = 1.0, ε=2.85, $\delta =10^{-5}$.

Model	Recall	F1 score
BERT	78.46%	78.26%
RoBERTa	80.56%	80.56%
BioBERT	75.52%	74.76%
ClinicalBERT	76.36%	76.25%
MentalBERT	80.28%	80.04%
MentalRoBERTa	81.82%	81.76%
DP-CARE (ours)	88.67%	78.08%

The results in Table 7 demonstrate that DP-CARE achieves performance that is competitive with or surpasses several domain-adapted transformer models. Our approach, which trains only a classifier on top of a frozen MentalBERT encoder using DP-AdamW, attains a recall of 88.67%—the highest among all compared models—while maintaining a competitive F1 score of 78.08%. This substantial gain in recall suggests that the noise introduced by DP-AdamW encourages the model to be more inclusive in identifying stressful posts. In mental health applications, where false negatives may have serious implications, this increase in recall can be particularly valuable. Although there is a slight drop in F1 compared to non-private fine-tuned models like MentalRoBERTa, DP-CARE offers a compelling trade-off: strong task performance, formal privacy guarantees, and significantly lower computational cost. These results support DP-CARE as a practical and ethically sound alternative to traditional fine-tuning in sensitive NLP applications.

### 5.9 Interpretation of the privacy budget

In practice, a privacy budget of ε≈3 represents a moderate and commonly accepted level of protection in applied differential privacy research. For comparison, prior work in healthcare and NLP applications involving sensitive tabular or textual data typically deploys DP mechanisms with ε values in the range of [1,8], depending on task complexity and utility requirements [25, 32]. Smaller values (e.g., ε<1) enforce very strong privacy but often lead to severe performance degradation due to the high amount of injected noise, whereas larger values (e.g., ε>8) substantially weaken privacy guarantees and offer diminishing returns. Thus, our chosen ε≈3 achieves a balanced trade-off between meaningful privacy protection and practical model utility, consistent with established practice in privacy-preserving healthcare NLP systems. Qualitatively, an ε of this magnitude implies that the model’s predictions remain statistically close to those obtained if any single training example were omitted, thereby providing plausible deniability for individual users while maintaining competitive accuracy.

### 5.10 Effect of different privacy budgets

Although we report results for a single privacy budget (ε≈2.85), the expected behavior of DP-CARE under different privacy levels follows well-established trends from differentially private optimization. Smaller privacy budgets (e.g., ε=1) require injecting more noise during training, which typically leads to reduced recall on subtle or minority classes, increased variability across random seeds, and overall lower F1 performance. Conversely, larger values of ε (e.g., 5 or higher) introduce less noise and allow the DP model to approach the predictive performance of the non-private baseline. Because DP-CARE applies privacy only to the classifier parameters while keeping the encoder frozen, the magnitude of these changes is primarily driven by the classifier’s ability to separate stress and non-stress examples under increasing noise levels. Exploring the quantitative effects of multiple privacy budgets is an important direction for future work.

## 6 Discussion

Our results demonstrate that DP-CARE, which trains only a lightweight classifier with DP-AdamW on top of a frozen MentalBERT encoder, can achieve competitive performance on the Dreaddit dataset while providing a formal differential privacy guarantee of ε≈3. Despite the added noise and stricter update constraints inherent to DP-AdamW, the model retains high classification accuracy and maintains a macro F1 score comparable to the non-private baseline. These findings suggest that privacy-preserving training can be effectively integrated into mental health NLP workflows without substantial utility degradation.

Interestingly, the DP-trained model shows an increase in and recall but a slight drop in F1 score relative to the non-private model. The increase in recall can be explained by the gradient noise introduced by DP-AdamW, which acts as a form of regularization that discourages overfitting to dominant lexical patterns. This regularization makes the classifier more sensitive to minority or borderline cases, leading to a higher proportion of true positives. In the mental-health detection context, this is a valuable property—prioritizing recall means the model identifies more users expressing stress or distress, even if this slightly lowers precision by flagging some ambiguous posts. Qualitative inspection further supports this trend: the DP model captures short, context-dependent expressions of stress (e.g., “I can’t keep doing this”) that the non-private model often misses. This observation aligns with prior findings that DP noise can improve generalization and fairness across underrepresented subpopulations.

One of the key strengths of DP-CARE is its minimal cost—both in computational and privacy terms. By freezing the base transformer model and training only a small classifier module, we drastically reduce the number of trainable parameters, resulting in significantly lower GPU memory usage and training time. This enables deployment in environments with limited computational resources, such as mobile devices or low-power clinical infrastructure. Moreover, because only the classifier is updated using DP-AdamW, the privacy budget is spent solely on a small subset of parameters, making it possible to achieve a moderate ε without the severe utility loss commonly seen in full-model DP training. This combination of low compute, low privacy cost, and high utility makes DP-CARE a practical and scalable solution for sensitive NLP tasks.

However, several limitations warrant further investigation. First, while our current setup demonstrates privacy-preserving training with strong utility on Dreaddit, additional experiments are needed to evaluate generalizability across other mental health datasets and tasks, such as multi-class classification or symptom severity prediction. Second, our current analysis does not include a detailed per-class breakdown or error analysis, which could offer further insight into the strengths and weaknesses of DP-trained models in this domain. Finally, while training only the classifier offers practical advantages, future work could explore full-model fine-tuning with differential privacy or partial unfreezing strategies to balance performance and privacy more flexibly.

Overall, our findings contribute to the growing body of work at the intersection of privacy-preserving machine learning and mental health NLP, and provide a practical path forward for building safer, ethical, and deployable models in this sensitive domain.

## 7 Generalizability beyond dreaddit

While our evaluation focuses on the Dreaddit dataset for binary stress detection, the DP-CARE design is readily applicable to a broader range of mental-health NLP tasks. Because DP-CARE trains only a lightweight classifier on top of a frozen MentalBERT encoder, the model inherits the encoder’s strong domain-specific representations, which have previously demonstrated robust transfer to related conditions such as depression, anxiety, and crisis detection. The class-specific patterns we observe under differential privacy—such as increased recall on the positive (stress) class accompanied by reduced precision on the negative class—are consistent with well-established effects of DP optimization and are therefore likely to generalize across other imbalanced mental-health datasets. Importantly, this classifier-only DP formulation reduces computational cost while preserving the flexibility to extend the approach to multi-class or multi-condition settings. Future work will explore cross-dataset and cross-domain evaluations to further assess DP-CARE’s generalizability.

## 8 Conclusion

We presented DP-CARE, a privacy-preserving framework for mental health text classification that trains a lightweight classifier on top of a frozen domain-specific encoder using DP-AdamW. Evaluated on the Dreaddit dataset, DP-CARE achieves strong predictive performance while providing a formal differential privacy guarantee of ε≈3. By freezing the encoder and restricting training to the classifier, our approach significantly reduces the computational burden and the privacy budget, making it highly suitable for real-world deployment in privacy-sensitive or resource-constrained settings.

Training classifiers with DP-AdamW atop frozen mental health language models represents a practical and ethical compromise. It provides a path forward for incorporating privacy-preserving principles into mental health NLP without sacrificing usability or requiring extensive computational resources. Our encouraging results support the feasibility of safe model sharing and low-cost deployment, which are critical for enabling responsible use of language technology in applications involving vulnerable populations.

Future work will explore extending DP-CARE to more diverse mental health tasks, including multi-label classification, and investigate partial fine-tuning strategies under DP constraints to further enhance performance while maintaining strong privacy guarantees.

## 9 Limitations

While DP-CARE presents a practical and efficient framework for privacy-preserving stress detection, several limitations must be acknowledged.

First, the model architecture is restricted to training only the classifier on top of a frozen transformer-based encoder. While this design minimizes the privacy budget and computational cost, it also limits the model’s ability to adapt to the nuances of the downstream task. A frozen encoder cannot learn dataset-specific linguistic patterns or contextual shifts present in the target domain. This trade-off may cap performance, especially in more complex or heterogeneous datasets. Future work could explore hybrid strategies, such as unfreezing the top layers of the encoder or integrating adapter modules trained under DP, to balance adaptability and privacy guarantees.

Second, our evaluation is limited to a single dataset—Dreaddit—which, although diverse in topic, focuses narrowly on binary stress classification in Reddit posts. This restricts the scope of our conclusions regarding the generalizability of the approach. The broader mental health NLP landscape includes multi-label settings (e.g., co-occurrence of anxiety and depression), temporal progression analysis, and real-world clinical note classification. Applying DP-CARE to a broader range of datasets and tasks would offer a more comprehensive understanding of its robustness and practical applicability.

Lastly, while we achieve a privacy budget of approximately ε≈3, interpreting this value in real-world, high-stakes mental health contexts remains challenging. What constitutes an “acceptable” level of privacy is context-dependent and may vary depending on the severity of data sensitivity, the deployment environment (e.g., clinical vs. consumer-facing app), and applicable regulations (e.g., GDPR, HIPAA). Thus, future research should not only refine technical performance under stricter privacy budgets but also engage with interdisciplinary stakeholders—such as ethicists, clinicians, and legal experts—to better calibrate privacy guarantees to practical risk thresholds.

## Data Availability

Publicly available datasets were analyzed in this study. This data can be found here: https://aclanthology.org/D19-6213.pdf.

## Footnotes

1 The dataset is publicly available http://www.cs.columbia.edu/∼eturcan/data/dreaddit.zip.

## Appendix

### 1 DP-CARE training procedure

For reproducibility, we include a concise overview of the DP-CARE training process using the Opacus library (Algorithm 1). The procedure applies differential privacy only to the classifier parameters while keeping the MentalBERT encoder frozen.

Algorithm 1 Training DP-CARE with Opacus **Require:** Frozen MentalBERT encoder, dataset *D*, privacy parameters (ε, δ), noise multiplier σ, max grad norm *C* 1: Initialize classifier parameters θ 2: Initialize DP optimizer (DP-AdamW) with (σ, *C*) 3: **for** epoch = 1 to 10 **do** 4:   **for** each minibatch b⊂D **do** 5:     Compute loss L(θ;b) 6:     Clip per-sample gradients to norm *C* 7:     Add Gaussian noise $N(0,\sigma^{2}C^{2}I)$ 8:     Update classifier parameters θ 9:   **end for** 10: **end for** 11: Compute total privacy cost (ε,δ) using the Opacus accountant

### 2 Reproducibility resources

To facilitate transparency and reproducibility, we provide scripts and configuration files used in all experiments. The repository includes:

- Preprocessing scripts for the Dreaddit dataset (train/validation/test splits).
- Model training scripts for both the non-private and DP-CARE classifiers (PyTorch + Opacus).
- Utility code for computing privacy budgets (ε,δ) and aggregating metrics across random seeds.
- A reproducibility YAML file specifying all hyperparameters and random seeds.

The full implementation and instructions for replicating our experiments are available at: https://github.com/JimKarpodinis/PrivacyInformedMentalBert
